# Zerumbone Promotes Cytotoxicity in Human Malignant Glioblastoma Cells through Reactive Oxygen Species (ROS) Generation

**DOI:** 10.1155/2020/3237983

**Published:** 2020-05-06

**Authors:** Mohammad Jalili-Nik, Mohammad Montazami Sadeghi, Elmira Mohtashami, Hamid Mollazadeh, Amir R. Afshari, Amirhossein Sahebkar

**Affiliations:** ^1^Department of Medical Biochemistry, Faculty of Medicine, Mashhad University of Medical Sciences, Mashhad, Iran; ^2^Student Research Committee, Faculty of Medicine, North Khorasan University of Medical Sciences, Bojnurd, Iran; ^3^Pharmacological Research Center of Medicinal Plants, Mashhad University of Medical Sciences, Mashhad, Iran; ^4^Department of Physiology and Pharmacology, Faculty of Medicine, North Khorasan University of Medical Sciences, Bojnurd, Iran; ^5^Natural Products and Medicinal Plants Research Center, North Khorasan University of Medical Sciences, Bojnurd, Iran; ^6^Halal Research Center of IRI, FDA, Tehran, Iran; ^7^Biotechnology Research Center, Pharmaceutical Technology Institute, Mashhad University of Medical Sciences, Mashhad, Iran; ^8^Neurogenic Inflammation Research Center, Mashhad University of Medical Sciences, Mashhad, Iran

## Abstract

Glioblastoma multiforme (GBM) is the most hostile tumor in the central nervous system. Unfortunately, the prognosis of GBM patients is poor following surgical interventions, chemotherapy, and radiotherapy. Consequently, more efficient and effective treatment options for the treatment of GBM need to be explored. Zerumbone, as a sesquiterpene derived from *Zingiber zerumbet* Smith, has substantial cytotoxic and antiproliferative activities in some types of cancer. Here, we show that exposure of GBM cells (U-87 MG) to Zerumbone demonstrated significant growth inhibition in a concentration-dependent manner. Zerumbone also induced apoptosis and caused cell cycle arrest of human GBM U-87 MG cells in the G2/M phase of the cell cycle. In detail, the apoptotic process triggered by Zerumbone involved the upregulation of proapoptotic Bax and the suppression of antiapoptotic Bcl-2 genes expression as determined by qRT-PCR. Moreover, Zerumbone enhanced the generation of reactive oxygen species (ROS), and N-acetyl cysteine (NAC), as an antioxidant, reversed the ROS-induced cytotoxicity of U-87 MG cells. The Western blot analysis suggested that Zerumbone activated the NF-*κ*B p65, which was partly inhibited by NAC treatment. Collectively, our results confirmed that Zerumbone induces cytotoxicity by ROS generation. Thus, the study raises the possibility of Zerumbone as a potential natural agent for treating GBM due to its ability to induce cytotoxicity.

## 1. Introduction

Glioblastoma multiforme (GBM), as the most lethal primary brain tumor, has an overall survival rate of about one year following diagnosis [1, 2]. GBM is characterized by increased proliferation and a lack of apoptosis [3]. Given extensive treatments, including maximal resection, radiation, or chemotherapy with the alkylating agent temozolomide (TMZ), patients of GBM typically have an abysmal prognosis [4]. Hence, this emphasizes the necessity of new treatment approaches in GBM patients. Various studies have proposed that apoptosis deregulation influences the balancing between cell proliferation and cell death, contributing to cell growth [5]. Besides, growth factors and cytokines, especially nuclear factor kappa B (NF-*κ*B), promote the generation of reactive oxygen species (ROS) in the cytotoxicity and development of cancer [6].

**Figure 1:**
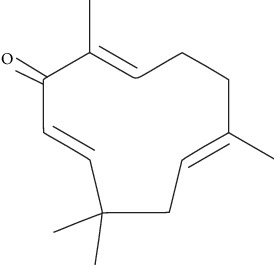
Chemical structure of Zerumbone (2,6,9,9-tetramethyl-[2E,6E,10E]-cycloundeca-2,6-10-trien-1-one, MW 218.33 g/mol).

Zerumbone (Figure 1), a natural crystalline cyclic sesquiterpene, is the main biological component in *Zingiber zerumbet* Smith rhizome, which is stated to have significant application potential in chemoprevention and chemotherapy approaches both *in vitro* and *in vivo* [7]. Numerous studies suggest that Zerumbone is an effective antiproliferative medication for the treatment of different cancer types such as colon, breast, cervical, and liver cancer and has selective effects on cancer cells compared to healthy cells [8–10]. Zerumbone has also been shown to suppress the growth of human colonic adenocarcinoma cell lines while being less effective in normal human dermal and colon fibroblasts [11]. Until now, however, the anticancer properties of Zerumbone have not been identified in GBM cancer studies. Numerous signaling pathways are involved in the antitumor effects of Zerumbone, including the NF-*κ*B regulation, induced nitric oxide synthase, and apoptosis [12, 13]. Its potential cytotoxic activity is owing to the versatile composition of *α*, *β*-unsaturated carbonyl, which plays an essential role in the compound associated with the most biologically active molecules [14].

A mechanistic study on the cytotoxic effects of Zerumbone on GBM cells (U-87 MG) was investigated. Initially, we found that Zerumbone induces cytotoxicity in GBM cells at different concentrations and attenuates tumor growth through the ROS mechanism. Consequently, Zerumbone promoted NF-*κ*B protein expression, which was inhibited by the approved ROS inhibitor (NAC). Also, we showed that Zerumbone caused cell cycle arrest at the G2/M phase, and Zerumbone-induced apoptosis could be initiated by the upregulation of the Bax/Bcl-2 ratio. Taken together, our outcomes indicate that Zerumbone is an encouraging player in GBM cell cytotoxicity that provides better options in the development of new therapies.

## 2. Materials and Methods

### 2.1. Chemicals and Reagents

Fetal bovine serum (FBS) and high-glucose Dulbecco's Modified Eagle's Medium (DMEM) were purchased from Gibco (Grand Island, NY, USA). Propidium iodide (PI), trypsin-EDTA, dimethyl sulfoxide (DMSO), MTT powder, and penicillin-streptomycin were bought from Sigma-Aldrich (St. Louis, MO, USA). The dichloro-dihydro-fluorescein diacetate (DCFDA)/H2DCFDA-cellular ROS detection assay kit and Zerumbone (extracted from *Zingiber officinale*) were obtained from Abcam (Cambridge, United Kingdom). N-acetyl-cysteine (NAC) was obtained from Cayman Chemical (Michigan, MI, USA). The annexin V-FITC early apoptosis assay kit and antibodies specific against NF-*κ*B p65 and beta-actin were prepared from Cell Signaling Technology (Beverly, MA, USA). The bicinchoninic acid (BCA), a protein assay kit, was bought from Pierce Co. (Pierce, Rockford, IL, USA). All other chemicals were purchased from Sigma-Aldrich (St. Louis, MO, USA) unless specified otherwise.

### 2.2. Cell Cultures

The U-87 MG malignant GBM cell line (NCBICode C531) and primary astrocyte cells (NCBICode C617) were obtained from the National Cell Bank of Iran (NCBI), Pasteur Institute (Tehran, Iran), and were cultured in DMEM with 10% FBS and antibiotics (100 *μ*g/mL streptomycin and 100 U/mL penicillin).

### 2.3. Cell Viability Assay

As previously described, a colorimetric MTT assay was used to assess cell proliferation [4, 5]. Briefly, U-87 cells were seeded in 96 wells (10^4^ per each well), and after overnight, the cells were incubated with various concentrations of Zerumbone (0–400 *μ*M) for 24 and 48 hours. Then, the MTT solution (phosphate buffer saline, 5 mg/mL) was added to each well, and in the following 3 hours, the formazan precipitate was disintegrated in DMSO. The absorbance at 570 and 620 nm (background) was measured on a Stat FAX303 plate reader. All the treatments were done in triplicate.

### 2.4. ROS Assay

This method was examined by the cellular ROS detection kit according to the manufacturer's instructions with minor modifications [5]. The 25 × 10^3^ U-87 MG cells were seeded and incubated overnight. After washing, the cells were incubated with 25 *μ*M of H_2_DCFDA solution for 30 minutes in the dark. Then the cells were rewashed and treated with Zerumbone (75 and 150 *μ*M) or NAC (10 mM) for 24 hours. The fluorescence was measured (Excitation/Emission: 485/535 nm) with the fluorescence plate reader FACScan (Becton Dickinson, San Jose, USA). tert-Butyl hydroperoxide (TBHP, 150 *μ*M) was used as a positive control. All treatments were done in triplicates.

### 2.5. Cell Cycle Analysis

The 7 × 10^5^ U-87 MG cells were treated with Zerumbone (18.75, 37.5, and 75 *μ*M) for 24 hours. Then, DNA content analysis was performed with PI staining as described [15].

### 2.6. Annexin V-FITC Assay

Apoptosis of GBM cells treated 24 hours with Zerumbone (18.75 and 37.5 *μ*M) was detected by an annexin V-FITC kit according to the manufacturer's instructions (Cell Signaling Technology, USA). Finally, flow cytometric analysis was conducted using a BD FACSCalibur™ flow cytometer (Becton Dickinson, Mountain View, CA, USA). Data analysis was done utilizing the software FlowJo® vX.0.7 (Tree Star, Ashland, OR, USA). All the treatments were done in triplicates.

### 2.7. RNA Analysis and Quantitative Reverse Transcription- (qRT-) PCR

Total RNA was extracted from the treated cells by Zerumbone (37.5 and 75 *μ*M) according to the manufacturer's instructions (Qiagen, Valencia, CA, USA), and qRT-PCR (with specific primers for GAPDH, Bax, Bcl-2, and p53 (Table 1)) was done as described [5].

### 2.8. Western Blot Analysis

GBM cells were seeded out, treated with Zerumbone (75 *μ*M), rinsed, and resuspended in RIPA lysis buffer. The 50 ng protein samples were quantified using a BCA assay and separated by 8-12% SDS-PAGE and transferred onto polyvinylidene difluoride (PVDF) membranes (Bio-Rad, HC, USA). The membranes were blocked with 5% nonfat milk in Tris-buffered saline- (TBS-) Tween overnight at room temperature and then incubated with the following specific antibodies, NF-*κ*B p65 (1 : 1,000) and beta-actin (1 : 1,000), at 4°C overnight followed by incubation with horseradish peroxidase-conjugated secondary antibody (1 : 3,000) at room temperature for 1 hour. Immune complexes were visualized using the Super Signal® West Femto (Thermo Fisher Scientific, Inc., Waltham, MA, USA) Western blotting kit as indicated by the manufacturer's directions. The relative expression was performed utilizing the ImageJ 1.52a software (NIH, Bethesda, Rockville, MD, USA) and then compared to the beta-actin protein.

## 3. Statistical Analysis

The experimental data were analyzed using software GraphPad Prism® 8.2.1 (GraphPad Software, San Diego, CA, USA). Data were expressed, and the mean ± standard error of the mean. The quantitative ratios of different groups were compared using the one-way analysis of variance followed by the Dunnett test. *p* < 0.05 was considered to indicate a statistically significant difference. All data were examined in triplicate against untreated control cells and collected from three independent experiments.

## 4. Results

### 4.1. Zerumbone Inhibits the Proliferation of U-87 MG Cells

**Figure 2:**
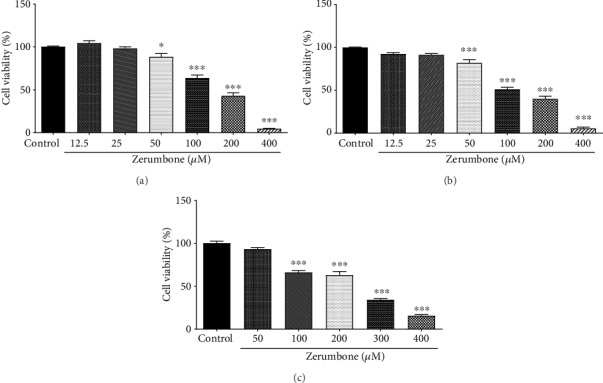
Dose- and time-dependent effects of Zerumbone on cell viability in U-87 MG cells following 24 (a) and 48 (b) hours treatment, and primary astrocyte cells following 24 hours' treatment (c). Cell viability was determined by the MTT assay. ^∗^*p* < 0.05 and ^∗∗∗^*p* < 0.001 versus the control group. Data are presented as the mean ± standard error of the mean (*n* = 8).

After the cells were treated with different concentrations of Zerumbone (12.5, 25, 50, 100, 200, and 400 *μ*M, *n* = 4) for 24 and 48 hours, the U-87 MG cell growth inhibition was recorded and is demonstrated in Figure 2. The outcomes of the MTT assay showed that Zerumbone mitigates the proliferation of U-87 MG cells concentration- and time-dependently. At a concentration of 100 *μ*M, Zerumbone inhibited the proliferation of U-87 MG cells by approximately 63% and 52% for 24 and 48 hours, respectively. Notably, the viability of primary astrocytes was not significantly affected by exposure to the equivalent concentrations of Zerumbone (half-maximal inhibitory concentration (IC_50_) > 200 *μ*M). The IC_50_ values for Zerumbone in U-87 MG cells were 150 and 130 *μ*M for 24 and 48 hours, respectively. In subsequent experiments, doses below the IC_50_ were therefore used.

### 4.2. Effects of Zerumbone on ROS Level

**Figure 3:**
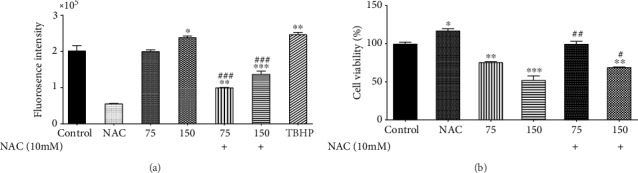
(a) Effects of Zerumbone on the ROS level on the GBM cells. Our data show that Zerumbone generates reactive oxygen species (ROS) levels in 24 hours. The cells were treated by Zerumbone (75 and 150 *μ*M) for 24 hours. A fluorimeter measured ROS levels. In the tert-Butyl hydroperoxide (TBHP) positive control (150 *μ*M) sample, the fluorescence intensity increased significantly compared to the control group. N-acetyl cysteine (NAC, 10 mM) decreased the Zerumbone-induced ROS generation at 24 hours after treatment, significantly (^###^*p* < 0.001 as compared with each group in the same concentration, ^∗^*p* < 0.05, ^∗∗^*p* < 0.001, and ^∗∗∗^*p* < 0.001 as compared with the control) (*n* = 4). (b) N-acetyl cysteine (NAC, 10 mM) combined with Zerumbone increased the viability of the U-87 MG cells at concentrations of 75 and 150 *μ*M 24 hours after treatment compared with each group in the same concentrations. Each column represents the mean ± standard error of the mean in the samples. (^##^*p* < 0.01 and ^#^*p* < 0.05 as compared with each group in the same concentration and ^∗^*p* < 0.05, ^∗∗^*p* < 0.001, and ^∗∗∗^*p* < 0.001 as compared with the control) (*n* = 4).

We determined ROS levels by fluorimeter to evaluate the role of ROS in Zerumbone-induced cytotoxicity (Epoch, BioTek® Instruments, Inc., USA). As Figure 3(a) shows, the treatment with Zerumbone (24 hours) in comparison with the control cells led to a significant and concentration-dependent elevation in the levels of ROS, resulting in oxidative damage of the GBM cells. However, ROS level elevation by Zerumbone at a concentration of ½IC_50_ was not remarkable. Besides, as shown in Figure 3(a), NAC (a ROS inhibitor, 10 mM) significantly diminished Zerumbone-induced ROS generation compared to the control group. Interestingly, our data show that NAC reversed the cell viability at 24 hours in Zerumbone-treated cells (Figure 3(b)). Hence, it is hypothesized that ROS is one of the main mechanisms of Zerumbone-induced cytotoxicity in GBM cells.

### 4.3. Zerumbone Induces Cell Cycle Arrest at the G2/M Phase in U-87 MG Cells

**Figure 4:**
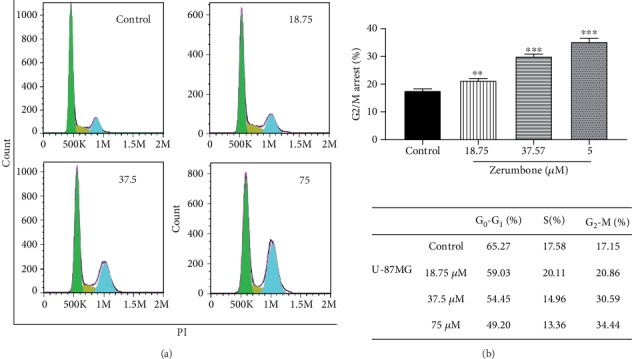
(a) Effect of Zerumbone (18.75, 37.5, and 75 *μ*M) on the cell cycle in U-87 MG cells following 24 hours' treatment. (b) Each column represents the mean ± standard error of the mean in the samples. Data presented are representative of three independent experiments. ^∗∗∗^*p* < 0.001 and ^∗∗^*p* < 0.01 versus the control group.

Analysis of the cell cycle by flow cytometry revealed that treatment with 18.75, 37.5, or 75 *μ*M Zerumbone enhanced the proportion of cells in the G2/M stage significantly (Figure 4, from 17.15 ± 0.40 to 20.86 ± 0.61, 30.59 ± 0.13, and 37.44% ± 0.54 following 18.75, 37.5, and 75 *μ*M of Zerumbone treatment, respectively). These findings suggest that Zerumbone caused a concentration-dependent arrest of the U-87 MG cell cycle at G2/M in lower concentrations of IC_50_.

### 4.4. Zerumbone Causes U-87 MG Cell Apoptosis

**Figure 5:**
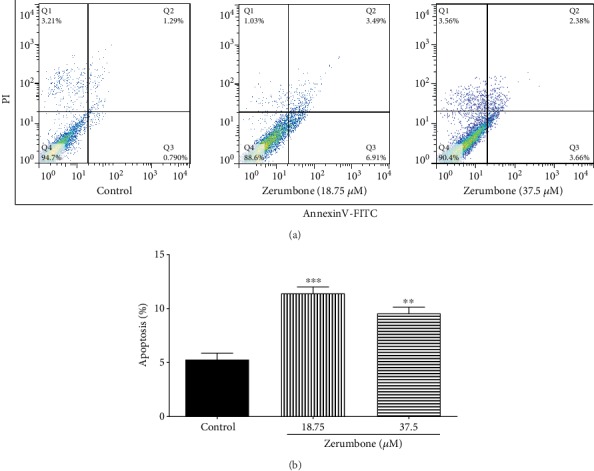
Flow cytometry analysis of Zerumbone-induced apoptosis in U-87 MG cells stained with annexin V-FITC/PI. Cells shown in the lower right (annexin V+/PI−) are undergoing apoptosis. Cells shown in the upper right (annexin V+/PI+) are undergoing necrosis. There was no difference between the concentrations of Zerumbone statistically. Each column represents the mean ± standard error of the mean in the samples (^∗∗^*p* < 0.001 and ^∗∗∗^*p* < 0.001 as compared with the control group) (*n* = 4).

GBM cells were cultured in 18.75 or 37.5 *μ*M Zerumbone for 24 hours, and the proportion of apoptotic U-87 MG cells was then determined using annexin V-FITC staining and flow cytometry. The percentage of apoptotic cells increased from 5.2 to 11.5 or 9.6% following 18.75 or 37.5 *μ*M Zerumbone treatment, respectively (Figure 5). These results showed that treatment with Zerumbone could induce apoptosis in GBM cells at lower IC_50_ levels.

### 4.5. The Effect of Zerumbone on Expression Levels of Apoptosis-Related Genes in U-87 MG Cells

**Figure 6:**
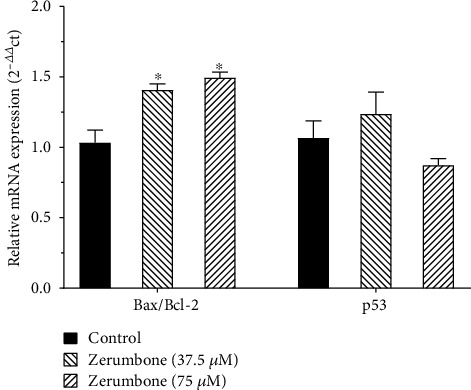
Cells were individually treated with 37.5 and 75 *μ*M Zerumbone for 24 hours, and then the cells were harvested for real-time- (RT-) PCR to determine the gene expression of the Bax/Bcl-2 ratio and p53. ^∗^*p* < 0.05 indicates a significant difference between control and treated cells.

The present study determined the impact of Zerumbone (37.5 and 75 *μ*M) on the expression of Bax, Bcl-2, and p53, as apoptosis-related genes by qRT-PCR. As shown in Figure 6, Zerumbone treatment increased Bax/Bcl-2 ratio genes at mRNA levels in U-87 MG cells compared to the control group. The p53 gene expression was not significant (*p* > 0.05).

### 4.6. Zerumbone Regulates NF-*κ*B p65 in U-87 MG Cells

**Figure 7:**
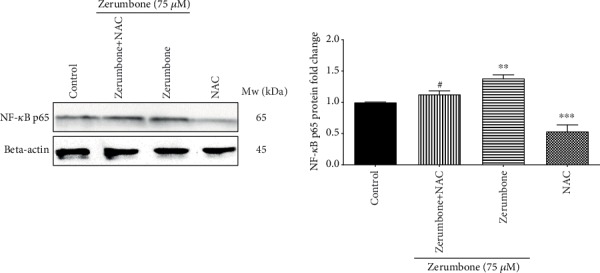
Effects of Zerumbone on the activation of NF-*κ*B p65. U-87 MG cells were treated with Zerumbone (75 *μ*M) for 1 hour, and the expression of NF-*κ*B p65 was detected by Western blot. The data were represented as mean ± standard error of the mean from three experiments. ^∗∗∗^*p* < 0.001 and ^∗∗^*p* < 0.01 versus the control group and ^#^*p* < 0.05 versus the Zerumbone-treated group.

In the action of chemotherapeutic drugs, the NF-*κ*B molecule plays a significant role. The present results showed that Zerumbone (75 *μ*M) activated NF-*κ*B significantly (Figure 7). This finding indicates that NF-*κ*B activation may play a critical upstream role in Zerumbone-mediated cytotoxicity and apoptotic activity of U-87 MG cells.

## 5. Discussion

Glioblastoma multiforme (GBM) in adolescents with relatively high recurrence levels is the most common form of primary brain tumor [3]. Despite the conventional treatments, such as chemo-radiation-therapy, the prognosis is low for GBM patients [5]. The molecular mechanisms controlling proliferation and recurrence to enhance the survival of GBM patients are therefore essential to study, as well as the design of a new approach to manage GBM patients with the inhibition of specific targets [2, 16]. Hence, throughout this research, the capability of Zerumbone, as a potential cytotoxic agent, to prevent GBM cancer cell growth *in vitro* has been assessed.

Zerumbone, a promising sesquiterpene derived from the medicinal herb *Zingiber zerumbet*, has been shown to exhibit a variety of biological and pharmacological effects [17]. Zerumbone has been found to present antioxidant, hepatoprotective, antitumor, and immunomodulatory activity [18]. Zerumbone treatment may induce apoptosis and inhibit tumor growth in a large number of tumor cells, such as breast cancer, colorectal cancer, lung cancer, and leukemia [19]. Notably, studies by Murakami et al. found that normal colon fibroblasts were not harmful compared with cancer cells once treated with Zerumbone at 50 *μ*M for 72 hours [20]. More notably, the consequent effects of Zerumbone on healthy astrocyte cells have been shown to be less harmful. Hoffmann et al. have demonstrated that Zerumbone (at specific concentrations) could induce a high intracellular redox potential which mitigates the tumor cell proliferation but not the normal cells. Hence, due to its regulatory role on intracellular redox [21], Zerumbone shows the ability to act as a potential cytoselective anticancer agent. Murakami et al. indicated that Zerumbone suppressed the initiation and development of skin tumors, but there is little research on the biological activity of Zerumbone on GBM [22, 23]. In line with the cytotoxic effects of Zerumbone, we observed that the tumorigenicity of U-87 MG cells was also inhibited by different concentrations of Zerumbone (IC_50_ of 150 and 130 *μ*M for 24 and 48 hours, respectively), concentration- and time-dependently, suggesting the inhibitory efficacy of Zerumbone on GBM cells. We then examined whether Zerumbone decreased the growth of cells by inducing the apoptosis of cells.

Uncontrolled growth and apoptosis failures are crucial elements in the progression and advancement of malignancy [24]. Therefore, the cell cycle and annexin V-FITC were analyzed to verify that Zerumbone-mediated cell death was due to apoptosis. Mechanistically, it has been indicated that Zerumbone has potential chemotherapeutic impacts in cervical and ovarian cancer cells through a G2/M phase cell cycle arrest [25, 26]. Examination of the cell cycle shows cells in the cell cycle G0/G1, S, and G2/M phases [27]. While investigating the mechanical action of Zerumbone, we found that Zerumbone could arrest the cell cycle in U-87 MG cells at the G2/M stage dose-dependently, and therefore, it reduced the proliferation of GBM cells. Studies have shown that multiple anticancer agents mediate G2/M phase arrest in cancer cells through different processes, such as tubulin polymerization disruption, regulated cyclin complexes, and interruption of the spindle assembly [28, 29]. Also, a study on breast cancer cells (MDA-MB-231 and MCF-7) showed that Zerumbone could lead to G2/M phase cell cycle arrest associated with Bax/Bak-mediated apoptosis and downregulation of cyclin B1, Ddk1, Cdc25C, and Cdc25B [30]. Hence, the increase in G2/M cells prevents the mitosis of cells [31]. Detection of the externalization of phosphatidylserine during apoptosis by annexin V-FITC staining further confirmed the apoptotic effect of Zerumbone.

Nevertheless, the result of the flow cytometry also showed cell death by late apoptosis and necrosis. Numerous findings have proven that apoptosis induction is associated with anti- and proapoptotic protein regulation. Bcl-2 (a notable protein that inhibits apoptosis) and Bax (a protein that promotes apoptosis) play a vital role in the apoptotic phase [32]. Several studies have shown that Zerumbone prevents the growth of the specific cancer cells through apoptosis induction by upregulating the Bax/Bcl-2 ratio [33]. Also, in a study, it has been shown that Zerumbone caused caspase-3 activation and poly(ADP-ribose) polymerase (PARP) production, contributing to GBM cell apoptosis [34]. In line with these findings, the results of RT-PCR detection showed that with the increase of Zerumbone concentration, the Bax/Bcl-2 ratio gradually increased as compared to the control group. The changes in the expression of these genes are characteristic of apoptosis and are thought to be key characters in human cells that induce apoptosis. The activation of apoptosis by Zerumbone was independent of p53, contrary to the expression of Bax and Bcl-2 genes since p53 levels showed no significant increase following Zerumbone treatment. Previous studies have shown that medications with apoptosis-inducing properties could reduce drug resistance in cancer cells [35]. We showed clearly that Zerumbone acts as an apoptosis-inducing agent and that Zerumbone can prove to become a crucial capable molecule to control GBM.

ROS plays a vital role in apoptosis induction and cytotoxicity [36]. In almost all cancers, high rates of ROS have been identified, promoting many aspects of tumor growth and development [37]. Several phytochemicals have reported intense anticancer activity by modifying the ROS level in cells [38]. The ROS levels in U-87 MG cells have been tested with fluorimetry to determine more precisely whether or not Zerumbone-induced cytotoxicity is related to changes in the ROS level. Exposure of U-87 MG cells to Zerumbone (at concentrations of IC_50_ and ½IC_50_) contributed to a marked increase in the rate of ROS.

Interestingly, NAC, as an antioxidant, decreased ROS generation induced by Zerumbone and reversed the cytotoxic effects of Zerumbone at concentrations of IC_50_ and ½IC_50_, indicating that cytotoxic effects of Zerumbone might be through the ROS mechanism. In further analysis, we demonstrated that Zerumbone markedly induces the NF-*κ*B protein expression, as evidenced by Western blot profiles, and this might be a vital factor of the induction of ROS, inducing cytotoxicity. In line with our findings, there are persuasive proofs that NF-*κ*B, as a transcription factor, is activated in several cell lines under various situations, including H_2_O_2_ and ROS [2, 39], which regulates multiple cellular processes such as proliferation, immunity system, development, inflammation, and apoptosis [40, 41]. The data showed that the noticeable event that occurred when U-87 MG cells were treated by Zerumbone is a marked increase in the level of NF-*κ*B factor. Interestingly, we observed a significant decrease in the NF-*κ*B protein level, when GBM cells were treated with Zerumbone plus NAC compared to the Zerumbone-treated group. Hence, the ROS generation serves as a critical driver for the NF-*κ*B activation.

**Figure 8:**
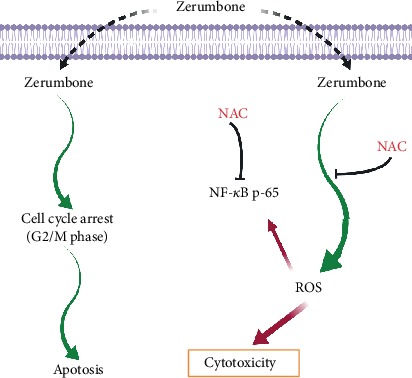
The suggested model of molecular signaling pathways after exposure to Zerumbone against GBM.

Collectively, the current study investigated Zerumbone cytotoxic potential in U-87 MG cells and found that in the G2/M phase, Zerumbone might induce cell death and cell cycle arrest. It was also noted that the antiproliferative activity of Zerumbone was followed by a corresponding increase in the level of ROS, which was reversed in U-87 MG cells via NAC treatment (Figure 8). Since GBM-induced cytotoxicity was mitigated by Zerumbone, even at low concentrations, the use of the natural product Zerumbone may be safer and may show reduced toxicity compared to other available treatments. These findings establish a framework for developing Zerumbone as a potential therapeutic alternative against GBM. Hence, apart from *in vitro* and *in vivo* findings, clinical trials may be conducted further to confirm the beneficial efficacy of Zerumbone in affected patients.

## Figures and Tables

**Table 1:** Primer sequences for PCR analysis of apoptotic-related genes.

Gene symbol	Gene name	Primers (5′ ⟶ 3′)	Accession number	Product length
Bax	Bcl-2-associated X protein	Forward: GGAGCTGCAGAGGATGATTG	NM_138761.4	100
		Reverse: CCAGTTGAAGTTGCCGTCAC		

Bcl-2	B-cell lymphoma 2	Forward: CTGAGGAGCTTTGTTTCAACCA	NM_000633.2	100
		Reverse: TCAAGAAACAAGGTCAAAGGGA		

P53	Tumor suppressor protein	Forward: ACCCTTGCTTGCAATAGGTG	NM_000546.5	100
		Reverse: AACAAAACACCAGTGCAGGC		

GAPDH	Glyceraldehyde-3-phosphate dehydrogenase	Forward: ACAACTTTGGTATCGTGGAAGG	NM_002046.7	101
		Reverse: GCCATCACGCCACAGTTTC		

## Data Availability

Data associated with this article are available from the corresponding author upon a reasonable request.

## Additional Points

*Highlights*. Zerumbone increases the level of ROS and induces arrest at the G2/M phase of the cell cycle in U-87 MG cells. An approved ROS inhibitor (N-acetyl cysteine (NAC)) could resist cell proliferation inhibition induced by Zerumbone, concentration-dependently. Zerumbone can prevent cell proliferation, maybe via ROS generation. The protein level of NF-*κ*B p65 induced by Zerumbone could be diminished by NAC.
